# Oxidative stress: from molecular studies to clinical intervention strategies

**DOI:** 10.3389/fmolb.2025.1638042

**Published:** 2025-09-04

**Authors:** Qing Gao, Linlin Jiang, Yuting Sun, Xuedong An, Wenjie Sun, Shanshan Tang, Xiaomin Kang, Xuefei Zhao, Zehua Li, Chenran Liu, Hangyu Ji, Fengmei Lian

**Affiliations:** ^1^ Guang’anmen Hospital, China Academy of Chinese Medical Sciences, Beijing, China; ^2^ Beijing University of Chinese Medicine, Beijing, China; ^3^ College of Traditional Chinese Medicine, Changchun University of Chinese Medicine, Changchun, Jilin, China

**Keywords:** oxidative stress, molecular mechanism, detection methods, antioxidants, diseases

## Abstract

The imbalance between the generation of free radicals and the body’s capacity to counteract their damaging effects on proteins, lipids, and nucleic acids is known as oxidative stress. Since it is essential for controlling many biological functions, this imbalance is intimately associated with the development and course of many diseases. In this study, we first outlined the submechanisms of oxidative stress, concentrating on the antioxidant system and reactive oxygen species. We also discussed common detection methods that can be beneficial for both clinical and scientific purposes. We examined prevalent diseases such as cardiovascular issues, diabetes, cancer, and neurodegenerative disorders to highlight the significant impact of oxidative stress. Additionally, we provided a list of common antioxidants to assist in clinical treatment and further exploration of underlying mechanisms. Our findings indicate that the molecular mechanisms of oxidative stress have been more thoroughly investigated, underscoring its scientific and clinical importance in understanding disease development and potential interventions. We propose that ongoing, in-depth research centered on oxidative stress could offer new insights for clinical interventions and mechanism exploration.

## 1 Introduction

An imbalance between the body’s oxidative and antioxidant mechanisms is a hallmark of oxidative stress (OS). Reactive oxygen species (ROS) is a broad term that encompasses oxygen-containing free radicals and peroxides that readily generate free radicals during oxygen metabolism. This includes superoxide anions (O_2_-), hydroxyl radicals (·OH), and hydrogen peroxide (H_2_O_2_). In humans, a primary source of ROS is the metabolic byproduct end of the respiratory chain located in the inner mitochondrial membrane. When mitochondrial function is impaired, enzyme complex IV in the inner mitochondrial membrane fails to produce water as it should, resulting in the production of O_2_-, which subsequently convert into ·OH and H_2_O_2_. This disruption can negatively affect the physiological functions of proteins, lipids, and even DNA (Viebahn-Haensler and León Fernández, 2024). Conversely, the human body has natural antioxidant systems, which include an enzymatic antioxidant system comprising superoxide dismutase (SOD), catalase (CAT), and glutathione peroxidase (GSH-Px), among others (Napolitano et al., 2021). Additionally, there is a non-enzymatic antioxidant system that includes compounds such as ergothioneine, vitamin C, vitamin E, glutathione, melatonin (MT), alpha-lipoic acid (ALA), carotenoids, and trace elements like copper, zinc, and selenium (Se). Consequently, oxidative stress typically arises from a general imbalance between oxidative and antioxidant systems.

## 2 Molecular mechanism of oxidative stress

### 2.1 Reactive oxygen species

ROS encompass O_2_- , ·OH , and H_2_O_2_. The primary source of ROS is the electron transport chain (ETC) located in the mitochondria (Mapuskar et al., 2024). In the respiratory chain, electron transmission is facilitated by four complexes and electron carriers on the inner mitochondrial membrane. This results in the production of H_2_O at complex IV and the creation of a gradient in proton concentration across the membrane, which promotes the synthesis of ATP. O_2_ may prematurely take up electrons from the ETC during this process, producing a small amount of O_2_-. (approximately 0.1%) (Mapuskar et al., 2017). In certain situations, such as aging, the ETC’s functionality may decline, leading to an excessive diversion of electrons from the ETC pathway, which then react with O_2_ to generate significant amounts of O_2_-, the precursors to most ROS (Nilsson and Tarnopolsky, 2019; Sohal, 1993). The disproportionation of O_2_- (either naturally or through the action of SOD) results in the formation of H_2_O_2_, which can be fully reduced to H_2_O or partially to ·OH, known to be among the most potent oxidants in nature (Turrens, 2003). Additionally, O_2_- then can quickly reacts with nitric oxide (NO) to form peroxynitrite (ONOO−). Intense physical activity can also lead to an excessive generation of ROS within the mitochondria (Powers et al., 2020). ·OH can inflict significant damage to lipids, proteins, and DNA, thereby disrupting the balance of various bodily systems due to oxidative stress. While ROS are frequently associated with “damage” and “destruction”, they also play crucial physiological roles in the human body. For instance, phagocytes utilize ROS generated by NADPH oxidase (NOX) to eliminate pathogens (Lambeth, 2004). Consequently, conditions that promote phagocyte proliferation, such as the inflammatory response, lead to the production of substantial amounts of ROS via NOX. Furthermore, non-phagocytic cells have been found to express NADPH oxidase (NOX4) specifically, which causes a considerable amount of ROS to be produced in a particular organ (Shiose et al., 2001; Geiszt et al., 2000). In summary, O_2_-, ·OH,and H_2_O_2_ are produced in significant amounts during aging, inflammation, and other external stimuli, causing harm to normal cells and contributing to the oxidative stress imbalance.

### 2.2 Antioxidant system

The human body’s antioxidant system consists of both enzyme-based and non-enzyme-based components. The enzyme antioxidant system includes SOD, CAT, and GSH-Px, among others. The non-enzyme antioxidant system comprises substances such as ergothioneine, vitamins C and E, glutathione, MT, ALA, carotenoids, and trace elements like copper, zinc, and Se.

#### 2.2.1 Enzymatic antioxidants

SOD plays a crucial role by converting O_2_- into H_2_O_2_, marking the initial step in the antioxidant process (Powers et al., 2020). While H_2_O_2_ is still quite toxic, it prevents the reaction between O_2_- and nitric oxide (NO) that would produce peroxynitrite (ONOO−). There are three types of SOD in the human body: manganese-dependent SOD (MnSOD) found in mitochondria, and copper-zinc-dependent SOD (CuSOD and ZnSOD) located in the cytoplasm (Hearn et al., 1999). Once H_2_O_2_ is formed, additional enzymes are needed to prevent the generation of more harmful ·OH. The primary enzymes responsible for this transformation in the human body are CAT and GSH-Px. CAT converts H_2_O_2_ into H_2_O and O_2_ using two H_2_O_2_ molecules without requiring extra electrons. In contrast, GSH-Px reduces H_2_O_2_ to H_2_O by utilizing additional electrons provided by reduced glutathione (GSH). Additionally, peroxiredoxin (Prx) is key component of the antioxidant system. Prx is a type of peroxidase, primarily functions to eliminate H_2_O_2_ from the body.

#### 2.2.2 Non-enzymatic antioxidants

There are numerous types of non-enzymatic antioxidants. Some non-enzymatic antioxidants exert their effects by participating in the catalytic reactions of various enzymes. For instance, trace elements such as copper and zinc are important components of SOD. Glutathione, on the other hand, reduces H_2_O_2_ to H_2_O by providing additional electrons. Some other non-enzymatic antioxidants possess their own reducing properties. Ergothioneine is a natural amino acid derived from plants and can accumulate in animals, which can effectively remove ·OH groups and prevent the formation of ·OH from H_2_O_2_. The fat-soluble vitamin E and the water-soluble vitamin C complement each other and jointly maintain the balance within cells. ALA is an organic compound that is both lipid-soluble and water-soluble which has significant electron affinity and the ability to react with free radicals, thus possessing strong antioxidant properties. MT,one of the hormones secreted by the pineal gland in the brain, has strong neuroendocrine immune regulatory activity and the ability to scavenge free radicals and act as an antioxidant. Carotenoids are the main source of vitamin A in the body and also possess antioxidant properties. Trx is one of the important redox regulatory molecules within cells. It helps maintain protein stability, thus preventing protein oxidation (Murphy, 2012).

## 3 Common detection methods

### 3.1 The principle of the assays

#### 3.1.1 Free radical scavenging kinetics

The oxygen-radical antioxidant capacity (ORAC) assay determines antioxidant capacity by monitoring the kinetics of oxidative fluorescence decay. Peroxyl radicals (ROO•), generated through controlled thermal decomposition of 2,2'-azobis (2-amidinopropane) dihydrochloride (AAPH) at physiological temperature (37 °C), oxidize a fluorescent probe (e.g., fluorescein). This oxidation induces progressive diminution of fluorescence intensity. Antioxidants interfere with this decay process by scavenging radicals, and their radical-scavenging capacity is quantified by calculating the net area under the time-dependent fluorescence decay curve (AUC) relative to the radical-exposed control (Miller et al., 1993; Ou et al., 2001). The total radical-trapping antioxidant parameter (TRAP) assay quantifies the capacity of antioxidants to delay fluorescence decay in a probe (such as R-phycoerythrin or dichlorofluorescein) under controlled peroxidative conditions initiated by AAPH-generated ROO•. Antioxidant capacity is determined by the length of the lag phase relative to a standard reference compound, typically Trolox (Prior et al., 2005; Ghiselli et al., 1995; Valkonen and Kuusi, 1997). The total oxidant scavenging capacity (TOSC) assay quantifies antioxidant capacity by measuring inhibition of α-keto-γ-methylthiobutyric acid (KMBA) oxidation to ethylene. ROO•generated through thermal decomposition of 2,2'-azobis (2-amidinopropane) AAPH, initiate this oxidation. Ethylene generation kinetics are monitored via headspace gas chromatography (HS-GC). Antioxidant activity is quantified by the reduction in area under the ethylene concentration-time curve relative to the radical-only control (Amorati and Valgimigli, 2015). The β-carotene bleaching assay quantifies antioxidant capacity by measuring inhibition of oxidative discoloration in a lipid peroxidation system. ROS generated during oxidation of unsaturated fatty acids, degrade β-carotene. This degradation manifests as decreased absorbance at 470 nm. Antioxidants retard the discoloration rate by suppressing lipid peroxidation chain reactions (Gulcin, 2020).

#### 3.1.2 Chemiluminescence quenching

Chemiluminescent assays quantify antioxidant activity by measuring the attenuation of chemiluminescence generated through reactions between ROS and specific probes such as luminol or lucigenin. Antioxidants reduce signal intensity either by competing with these probes for oxidants (notably H_2_O_2_) or by quenching generated radicals including O_2_- and hydroxyl radical (OH·) (Shahidi and Zhong, 2015; Shivakumar and yogendra kumar, 2018). Photochemiluminescence assay quantifies antioxidant activity by monitoring O_2_- radical scavenging capacity. A mercury lamp (λ = 351 nm) excites a photosensitizer to generate O_2_- radicals. Antioxidant activity is determined by the sample’s ability to scavenge these radicals, with results expressed as Trolox equivalents based on the chemiluminescent intensity of residual radicals (Neculai et al., 2023).

#### 3.1.3 Reducing power of single electron transfer

The Folin-Ciocalteu assay quantifies total phenolic content (TPC) through a single electron transfer (SET) mechanism in alkaline medium. Phenolic compounds reduce the phosphomolybdate complexes present in the Folin-Ciocalteu reagent, forming a blue chromophore with maximum absorbance at 765 nm. This absorbance is directly proportional to phenolic concentration (Singleton et al., 1999; Shahidi and Zhong, 2015). The cupric reducing antioxidant capacity (CUPRAC) assay quantifies antioxidant activity through the reduction of Cu^2+^-neocuproine to Cu^+^-neocuproine under near-neutral conditions (pH 7.0). This reduction generates an intense yellow chromophore exhibiting maximum absorbance at 450 nm (Apak et al., 2022; Gulcin, 2008; Tutem et al., 1991). The ferric reducing antioxidant power (FRAP) assay measures the reduction of the ferric 2,4,6-tripyridyl-s-triazine complex [Fe^3+^-(TPTZ)_2_]^3+^ to the blue ferrous complex [Fe^2+^-(TPTZ)_2_]^2+^ by antioxidants in acidic medium (pH 3.6), monitored by absorbance increase at 593 nm (Benzie and Strain, 1999).

#### 3.1.4 Stabilization of free radical scavenging

The 1-diphenyl-2-picrylhydrazyl (DPPH) radical scavenging assay quantifies antioxidant capacity by measuring the reduction of the stable DPPH• radical through hydrogen atom transfer (HAT) or electron transfer (ET) mechanisms. This reaction induces decolorization from purple to yellow, characterized by a decrease in absorbance at 517 nm, which is monitored spectrophotometrically (Brand-Williams et al., 1995). The trolox-equivalent antioxidant capacity (TEAC) assay quantifies antioxidant capacity by measuring the decolorization of the pre-formed blue-green ABTS^+^• radical cation (λ_max_ = 734 nm) following radical scavenging; the reduction in absorbance is measured spectrophotometrically and expressed as Trolox equivalents (Prior et al., 2005).

### 3.2 Advantages and limitations of the assays

#### 3.2.1 ORAC

The ORAC assay facilitates high-throughput screening through microplate-based parallel analysis. This method utilizes biologically relevant ROO• generated from 2,2'-azobis (2-amidinopropane) AAPH and employs AUC quantification to provide a comprehensive kinetic assessment of antioxidant activity. The assay accommodates both hydrophilic and lipophilic antioxidants; the latter requires solubility enhancers such as randomly methylated β-cyclodextrin (RMCD). However, several limitations exist. Strict control of buffer conditions (pH 7.4) is essential due to the pH sensitivity of the fluorescein probe. Furthermore, AUC integration inherently combines kinetic and stoichiometric data, potentially complicating interpretation. The relatively low radical reactivity of fluorescein may lead to underestimation of potent antioxidants with rapid reaction kinetics. Finally, precise temperature maintenance at 37 °C is critical throughout the assay procedure (Ou et al., 2001; Huang et al., 2002; Shivakumar and Yogendra kumar, 2018).

#### 3.2.2 TRAP

A key advantage of this assay is its sensitivity to all known chain-breaking antioxidants, with established clinical correlations in various disease states (Prior et al., 2005). However, significant limitations include operational complexity, time-consuming procedures requiring specialized expertise, and reliance on lag phase quantification. This reliance fails to capture the activity of antioxidants lacking distinct lag phases and disregards post-lag phase contributions. Furthermore, stability issues associated with oxygen electrodes further compromise reproducibility (Rice-Evans and Miller, 1994; Somogyi et al., 2007).

#### 3.2.3 TOSC

The TOSC assay quantifies antioxidant capacity by measuring the integrated inhibition of ethylene formation—determined via AUC analysis—during KMBA oxidation. This method eliminates interference from colored matrices through headspace gas chromatographic detection (Winston et al., 1998). However, the approach suffers from significant limitations: a time-consuming analytical workflow requiring multiple chromatographic runs per sample, and compromised mechanistic interpretability due to the AUC-based evaluation. This integration method inherently fails to distinguish between kinetic reactivity and stoichiometric parameters.

#### 3.2.4 β-Carotene bleaching assay

This method enables rapid screening of lipid-soluble antioxidants in emulsion systems. However, it exhibits significant limitations including quantification inaccuracies, limited reproducibility, and complex reagent preparation. Furthermore, measurements are susceptible to interference from pH fluctuations, temperature variations, and solvent effects, compromising reliability for absolute quantification (Alam et al., 2013; Apak et al., 2022).

#### 3.2.5 Chemiluminescence assay

This assay offers exceptional sensitivity for ROS detection through chemiluminescent probes such as luminol and lucigenin, enabling quantification of antioxidant activity via light emission quenching. Its versatility permits application across diverse oxidant-catalyst systems. Key limitations, however, include dependence on specialized instrumentation (e.g., luminescence photometers), susceptibility to interference from catalysts or enhancers, and pH-dependent mechanistic variations in the luminol reaction (Shahidi and Zhong, 2015).

#### 3.2.6 Photochemiluminescence assay

The photochemiluminescence assay enables precise quantification of O_2_- scavenging capacity through controlled photochemical generation and luminescence detection. Utilizing physiologically relevant O_2_-, this approach mimics oxidative stress conditions with high sensitivity (Chiefa et al., 2024). However, limitations include a restricted quantification range where undiluted samples (e.g., T96/F96 concentrations) inhibit calibration curve fitting, necessitating serial dilution (1:10–1:200) for accurate measurement. Additionally, reliance on specialized instrumentation (e.g., Photochem systems, Analytik Jena) and proprietary reagent kits constrains methodological accessibility.

#### 3.2.7 Folin-ciocalteu

This assay demonstrates operational simplicity, high reproducibility, and sensitivity, making it broadly applicable for estimating the reducing capacity of plant and food extracts. Its commercial availability further enhances practical utility (Singleton and Rossi, 1965; Prior et al., 2005). Notable limitations include non-specificity: numerous non-phenolic reducing agents (e.g., ascorbic acid, proteins, reducing sugars, and certain inorganic ions) react, leading to overestimation of TPC. Additionally, it is unsuitable for lipophilic antioxidants and exhibits significant pH- and temperature-dependence, requiring strict experimental control (Singleton et al., 1999; Prior et al., 2005; Shahidi and Zhong, 2015). The principles, advantages, and limitations of common detection methods are summarized in Table 1.

**TABLE 1:** Common detection methods and their principles, advantages, and limitations.

Grouping criteria	Assay	Principle	Advantages	Limitations
Free radical scavenging kinetics	ORAC	The ORAC assay quantifies antioxidant capacity by measuring peroxyl radicals ROO•, AAPH-derived at 37 °C) oxidizing fluorescent probes (e.g., fluorescein), causing fluorescence decay. Antioxidant activity is calculated as the net area under the fluorescence decay curve (AUC) relative to control	high-throughput screening Biologically relevant ROO• AUC kinetic integration Hydrophilic/lipophilic compatibility	Fluorescein pH sensitivity Kinetic/stoichiometric confusion High antioxidant content underestimates risks Temperature-dependent
	TRAP	The TRAP assay quantifies antioxidant capacity by measuring the delay (lag phase) in AAPH-ROO•-induced fluorescence decay of probes (e.g., R-phycoerythrin) relative to Trolox under controlled peroxidative conditions	Sensitive to chain-breaking antioxidants	Time-consuming operation Requires expertise Oxygen electrode instability Rely solely on the lag phase
	TOSC	TOSC quantifies antioxidant capacity by inhibition of ethylene formation from KMBA oxidation by ROO•, measured as reduced AUC via headspace GC.	Quantifies total antioxidant capacity Colored matrix interference-free detection	Time-consuming operation Weak chemical significance
	β-Carotene Bleaching	The β-carotene bleaching assay quantifies antioxidant capacity by inhibition of lipid peroxidation-induced oxidative bleaching. ROS generated during lipid oxidation degrade β-carotene, decreasing absorbance at 470 nm; antioxidants retard bleaching by scavenging radicals and breaking chain reactions	Rapid, straightforward screening tool	Quantification inaccuracies Low reproducibility Complex reagent preparation Susceptible to pH/temperature/solvent interference
Chemiluminescence quenching	Chemiluminescence	This detection method quantifies the activity of antioxidants by measuring the inhibitory effect of light emission produced during the reaction of ROS with chemiluminescent reagents such as luminol and lucigenin	High sensitivity Versatility	Requires specialized luminometer Potential interference from catalysts/enhancers Luminol mechanism pH-dependent
	Photochemiluminescence Assay	This assay quantifies O_2_•^-^scavenging capacity: a mercury lamp (λ = 351 nm) excites a photosensitizer to generate radicals; antioxidant activity is measured via reduced chemiluminescence intensity and expressed as Trolox equivalents	High sensitivity	Restricted quantification range Instrument dependency
Reducing power of single electron transfer	Folin-Ciocalteu	Quantifies total phenolic content via ET reduction of phosphomolybdate/phosphotungs-tate reagent in alkaline medium, forming blue chromophore (λmax = 765 nm)	Simple, reproducible, sensitive Widely applied	Lack of specificity Unsuitable for lipophilic antioxidants Affected by PH and temperature
	CUPRAC	The CUPRAC assay quantifies antioxidant activity via reduction of Cu^2+^-neocuproine to Cu^+^-neocuproine at pH 7.0, forming a chromophore measured at 450 nm	Stability and linearity of the reagent Hydrophilic/lipophilic compatibility Thiol-specific detection	Solvent Dependence for Lipophilics Slow Reaction Kinetics
	FRAP	The FRAP assay quantifies antioxidant power via reduction of Fe^3+^-TPTZ to blue Fe^2+^-TPTZ at pH 3.6, measured by absorbance increase at 593 nm	Simple, fast, cost-effective Amenable to automation Wide application	Ignores thiols/carotenoids Slow-reacting polyphenols underestimated at short times Pro-oxidant Fe^2+^generation
Stabilization of free radical scavenging	DPPH	The DPPH assay quantifies antioxidant capacity via HAT/ET-mediated reduction of the DPPH• radical, inducing purple-to-yellow decolorization measured spectrophotometrically at 517 nm	Simple, rapid, inexpensive Commercially available radical Extensively applicable to pure compounds and extracts	Spectral interference from sample chromophores Poor aqueous solubility of DPPH Variable and prolonged reaction kinetics
	TEAC	The TEAC assay quantifies antioxidant capacity by spectrophotometric measurement (734 nm) of ABTS^+^• radical decolorization, expressed as Trolox equivalents	Simplicity and convenience High-throughput screening capability Utilization of stable ABTS^+^• radical	Failure to distinguish kinetics from stoichiometry Reliance on single-point measurements Arbitrary time dependency

#### 3.2.8 CUPRAC

The CUPRAC assay possesses key advantages, including reagent stability, linearity, and applicability to both hydrophilic and lipophilic antioxidants. Notably, it effectively detects thiol-based antioxidants such as glutathione (Gulcin et al., 2007; Apak et al., 2022; Karaman et al., 2009). However, the method exhibits marked solvent-dependent variations in measured antioxidant capacity, particularly for lipophilic compounds (e.g., BHT and naringenin). Furthermore, suboptimal reaction kinetics under standard conditions have been documented for certain antioxidants (Celik et al., 2010).

#### 3.2.9 FRAP

FRAP assay offers advantages including simplicity, cost-effectiveness, robustness, and applicability to diverse matrices such as biological fluids, foods, and plant extracts (Benzie and Strain, 1999; Prior et al., 2005). However, the assay is constrained by several limitations. Its exclusive reliance on an ET mechanism precludes the assessment of HAT-based chain-breaking antioxidant activity or specific radical scavenging capacity. Furthermore, FRAP cannot accurately quantify thiol-containing antioxidants (e.g., glutathione) or carotenoids due to their poor interaction with ferric ions. Slow-reacting polyphenols, such as caffeic acid and quercetin, require extended reaction times for precise measurement, potentially leading to underestimation under standard protocols. Additional constraints include the potential for compounds with redox potentials higher than the Fe^3+^/Fe^2+^ couple to overestimate values, and interference from sample chromophores absorbing at 593 nm (e.g., biliverdin), causing overestimation. Moreover, the acidic assay conditions (pH 3.6) can precipitate proteins (e.g., milk casein) and generate pro-oxidant Fe^2+^ ions, which may propagate radical chain reactions (Benzie and Strain, 1999; Pulido et al., 2000; Pulido et al., 2003; Ou et al., 2002a; Ou et al., 2002b; Prior et al., 2005; Somogyi et al., 2007).

#### 3.2.10 DPPH

The DPPH assay offers several advantages, including simplicity, speed, cost-effectiveness, ease of use (owing to the commercial availability of the stable radical), and broad applicability to both pure compounds and extracts (Gulcin et al., 2007; Shahidi and Zhong, 2015). However, limitations include potential spectral interference from sample chromophores, such as anthocyanins, and the poor aqueous solubility of the DPPH radical. Additionally, the reaction kinetics are often inconsistent and prolonged (Brand-Williams et al., 1995; Bondet et al., 1997; Yamauchi et al., 2024).

#### 3.2.11 TEAC

The TEAC assay provides advantages of simplicity and convenience for high-throughput antioxidant screening through its utilization of the relatively stable ABTS^+^• radical cation. However, this method relies on single-point measurements (typically recorded at a fixed time, e.g., 5 min), which fail to differentiate between reaction kinetics (rate constants) and stoichiometry (number of radicals trapped per molecule). Consequently, the results become dependent on the arbitrarily selected measurement time point (Re et al., 1999).

### 3.3 Related applications

#### 3.3.1 ORAC

In a 12-month clinical trial, Donato Di Pierro et al. investigated oxidative stress’s consequences using the ORAC assay and found that coenzyme Q10 may significantly reduce oxidative stress-related damage in RTT erythrocytes (Di Pierro et al., 2020). In a 48-h randomized controlled trial, Angela R Hillman et al. reported no impact of acute Montmorency sour cherry intake on oxidative capacity after testing 48 participants with the ORAC method (Hillman and Uhranowsky, 2021). Ariko Umezawa et al. discovered a positive correlation between cholesterol efflux capacity and ORAC in a randomized, parallel-controlled clinical trial that examined cholesterol efflux after a 6-month Japanese diet (Umezawa et al., 2022). Carolina Fredes et al. investigated two of Chile’s southernmost berries, murtha and calamansi, and identified them as fruits with high ORAC values and natural antioxidant properties (Fredes et al., 2020). Additionally, Samia Elbahnaswy et al. suggested that astaxanthin could be a beneficial agent for preventing various oxidative stress-related diseases in aquatic animals, making it a promising candidate (Elbahnaswy and Elshopakey, 2024).

#### 3.3.2 TRAP

Marge Kartau et al. conducted a study analyzing TRAP and UA levels in 112 multiple sclerosis patients, discovering a positive correlation between UA and TRAP levels, with men exhibiting higher TRAP values than women (Kartau et al., 2024). Fatemeh Toorang et al. found in a large clinical study that diets rich in antioxidants, such as FRAP or TRAP, lowered the risk of developing head and neck cancer (HNC) and its subtypes (Toorang et al., 2023). G. Godoy et al. concluded from an experiment on plasma non-enzymatic antioxidant capacity in rats subjected to intense swimming that the TRAP assay is the most effective method for evaluating plasma non-antioxidant capacity following vigorous exercise (Kartau et al., 2024). The most sensitive assay for plasma non-antioxidant capacity post-exercise was identified (Godoy et al., 2022). Walter Sepúlveda-Loyola et al. utilized the TRAP assay in a clinical study to assess antioxidant capacity, finding a connection between COPD patients’ sarcopenia and oxidative stress (Sepúlveda-Loyola et al., 2021).

#### 3.3.3 TOSC

Mohamed Dellali and colleagues found a substantial rise in total oxygen radical scavenging capacity (TOSC) after 7 days of exposure to two concentrations of benzo [a]pyrene (B [a]P) (100 and 300 μg/L) in the digestive glands, with the lowest concentration showing the most significant increase (Dellali et al., 2021). The non-alcoholic steatohepatitis (NASH) model exhibited a significantly lower antioxidant capacity compared to the NO-NASH model, which is characterized by a dietary pattern rich in polyphenols, vitamins, and fiber, similar to the Mediterranean diet (Vitale et al., 2022).

#### 3.3.4 β-Carotene bleaching assays

Fatimata Nea et al. assessed the antioxidant capacity of Lantana rhodesiensis Moldenke extracts using DPPH, FRAP, and β-carotene bleaching tests, revealing that leaf extracts had higher antioxidant activity than stem and root extracts, likely due to a higher presence of polyphenols, including flavonoids. Positive linear correlations were found between phenolic content (total polyphenols, including flavonoids and tannins; and total flavonoids) and the antioxidant activity of all extracts (Nea et al., 2021). The antioxidant potential of hesperidin-related substances was assessed by Hyo-Jun Lee et al. using both DPPH and β-carotene bleaching methods, finding that all tested compounds exhibited antioxidant activity in a concentration-dependent manner, but to differing degrees (Lee et al., 2024). Nyayiru Kannaian reported significant antioxidant activity in coconut cotyledons as measured by DPPH, FRAP, NO, and β-carotene bleaching assays (Nyayiru Kannaian et al., 2020). Maria García-Nicolás et al. established a correlation between citrinin content and antioxidant activity as measured by the β-carotene bleaching assay (García-Nicolás et al., 2023). According to a study by Zhuang Y et al.acylated pectin showed enhanced antioxidant activity in both the DPPH and β-carotene bleaching assays, along with notable antimicrobial properties (Zhuang et al., 2022).

#### 3.3.5 Chemiluminescent assay

Na Wu et al. developed a novel chemiluminescence platform utilizing semiconducting polymer nanoparticles-manganese (SPN-MnVII) for detecting total antioxidant capacity in the urinary samples of mice with diabetes (Naderi et al., 2023).

#### 3.3.6 Photochemiluminescence assay

Mihaela Multescu and colleagues utilized the photochemiluminescence assay (PCL) along with methods such as DPPH, ABTS, Ferric Reducing Antioxidant Power (FRAP), and Copper Ion Reducing Antioxidant Capacity (CUPRAC) to assess the total antioxidant capacity (TACO) of vegetable oils sourced from the vegetable oil industry. They found a strong positive correlation between the fat-soluble antioxidant capacity (ACL) of 14 different by-products (including flour, crude flour, and hulled) measured by PCL and the other antioxidant activity methods (Multescu et al., 2022). Neculai AM and others evaluated the antioxidant capacity of oils from the vegetable oil industry using the DPPH radical scavenging assay and photochemiluminescence, discovering a strong link between antioxidant activity and the presence of phenolic compounds in the maceration solution (Neculai et al., 2023). Rajagukguk YV and colleagues noted a robust relationship between antioxidant activity and overall phenolic content (measured by ORAC, DPPH, ABTS, and PCL), with antioxidant activity declining as total phenolic content decreased over 2 months of storage (Rajagukguk et al., 2022).

#### 3.3.7 Folin-ciocalteu

Haldar et al. found that consuming (poly)phenol-rich curry, evaluated through the urinary Folin-Ciocalteu assay, improved glucose regulation in 20 healthy men (Haldar et al., 2019). In older populations from the PREDIMED and InCHIANTI studies, individuals with higher urinary (poly) phenol excretion exhibited better cardiovascular health indicators, including glucose and triglyceride levels, blood pressure, and body weight (Guo et al., 2016). Laveriano et al. reported that urinary polyphenols correlated with improved cardiovascular profiles in a Spanish adolescent group, with boys showing a stronger correlation than girls (Laveriano-Santos et al., 2020). Arancibia et al. demonstrated that urinary total (poly) phenol excretion, measured by the Folin-Ciocalteu assay, could serve as a reliable biomarker for an anti-inflammatory diet, particularly in females, supporting the inverse relationship between total polyphenol intake and inflammation (Arancibia-Riveros et al., 2023). Motto AE et al. conducted *in vivo* studies on the anti-hyperlipidemic effects of total extracts and supernatants from Anogeissus leiocarpus using a fructose overload assay in ICR mice, assessing total polyphenols and flavonoids through colorimetric assays with Folin-Ciocalteu reagent and aluminum chloride. They found that the extracts exhibited strong antihyperlipidemic and antioxidant properties, rich in polyphenols, which could be beneficial for treating diabetes-related macrovascular complications (Motto et al., 2021). Mamri S et al. employed the Folin-Ciocalteu reagent to evaluate total phenolic content in androgynous extracts, while the aluminum chloride method was used for total flavonoid content. Phytochemicals were quantified and identified using HPLC-DAD, and *in vitro* α-amylase inhibition tests indicated that C. sativus stamens effectively lower postprandial blood glucose levels (Mamri et al., 2024).

#### 3.3.8 CUPRAC

Milena Polumackanycz and colleagues conducted a thorough investigation into the antioxidant capacity of common and lemon verbena leaves using DPPH, ABTS^+^•, FRAP, and CUPRAC methods. They found that lemon verbena exhibited greater antioxidant activity than common verbena, which correlated positively with its phenolic content (Polumackanycz et al., 2022b). Leyla Polat Kose and her team also explored the antioxidant capacity of various phytolignans and mammalian lignans through ABTS^+^•, DPPH, CUPRAC, and FRAP, discovering that phytolignans, as secondary metabolites in plants, demonstrated relatively high antioxidant activity, while enterodiols and enterolactones showed weaker activity compared to phytolignans and standard antioxidants (Polat Kose and Gulcin, 2021). Mine Aydın Kurç et al. assessed the hexane extract of *Cirsium vulgare* using TEAC, FRAP, CUPRAC, a β-carotene bleaching assay, and O_2_- scavenging activity measurements, revealing that the methanolic extract had the highest total flavonoid content and antioxidant capacity (Aydın Kurç et al., 2023). Milena Polumackanycz et al. compared the antioxidant capacity of aqueous and hydro-methanolic extracts using DPPH, ABTS^+^•, FRAP, and CUPRAC, finding that the hydro-methanolic extract of R. rosea was richer in phenolic compounds and exhibited superior antioxidant and neurobiological activities compared to the aqueous extract (Polumackanycz et al., 2022a). Bailey R. Meyer and colleagues evaluated the antioxidant capacity of 15 commercially available green teas using CURPAC and ORAC, discovering that matcha, gunpowder, and bagged green teas had higher total phenolic content and stronger CUPRAC and ORAC antioxidant capacities compared to other teas (Meyer et al., 2023). Yara Salem et al. utilized DPPH, CURPAC, and FRAP to assess the antioxidant capacity of seeds from Obeidi, Asswad Karech, Marselan, Syrah, and Cabernet Franc, finding that Marselan had the highest total phenolic and proanthocyanidin content, along with the greatest antioxidant activity (Salem et al., 2022).

#### 3.3.9 FRAP

Paiva et al. detailed the preparation and findings of antioxidant studies on peptide fractions derived from protein hydrolysates of Spirulina muricata, with Fr3 showing the highest FRAP activity. The relevant applications of common detection methods are shown in Table 2. Additionally, Fr3 demonstrated significant angiotensin I-converting enzyme (ACE) inhibitory activity (Paiva et al., 2017). Kimatu et al. investigated the antioxidant activity of protein hydrolysates from Agaricus bisporus using DPPH and FRAP assays, finding that all hydrolysates and peptide fractions exhibited a concentration-dependent increase in iron-reducing antioxidant capacity, with trypsin hydrolysates showing the highest FRAP (Kimatu et al., 2017). Antioxidant activity was assessed using DPPH, ABTS, FRAP, and CUPRAC, confirming that exposure to ionizing radiation did not alter the chemical structure or antioxidant properties of the tested flavonoids (Rosiak et al., 2023).

**TABLE 2:** Common detection methods and their applications.

Assay	Aim of the study	Objects of study	Main findings	References
ORAC	To evaluate the effects of commonly used antioxidant supplements on erythrocyte energy metabolism and oxidative status in healthy adults	11 RTT patients	CoQ10 may considerably reduce the harm that oxidative stress causes to RTT erythrocytes	Di Pierro et al., 2020
ORAC	To compare the effects of different dosing regimens and formulations of anthocyanin-rich tart cherry supplements on inflammatory markers and antioxidant capacity in healthy adults	48 healthy volunteers	Oxidative capability is unaffected by the acute consumption of Montmorency sour cherries	Hillman et al., 2021
ORAC	To examine the impact of the Japan Diet on HDL cholesterol efflux capacity and its association with serum antioxidant concentrations	98 Japanese patients with dyslipidemia	It is believed that ORAC and cholesterol efflux capability are favorably connected	Umezawa et al., 2022
ORAC	To clarify the nutraceutical value of murta and calafate berries as functional food candidates	Murta and calafate	Fruits with a high ORAC and potential natural antioxidant sources are murta and calafate	Fredes et al., 2020
ORAC	To explore the molecular mechanisms underlying the nutritional and health benefits and ecological value of astaxanthin as a feed additive	Astaxanthin	In aquatic animals, astaxanthin may be a promising treatment option for a number of oxidative stress-related illnesses	Elbahnaswy et al., 2024
TRAP	To evaluate MS patients’ plasma antioxidant potential using the TRAP assay and examine its usefulness as an MS disease biomarker	112 MS patients	There were notable differences in TRAP levels between the sexes, with men having greater TRAP values than women, and UA was positively connected with TRAP values	Kartau et al., 2024
TRAP	To investigate the association between dietary total antioxidant capacity and HNC risk, while evaluating potential interactions with established HNC risk factors	876 HNC patients and 3,409 healthy controls	The risk of HNC and its subtypes may be reduced by eating a diet high in antioxidants, such as FRAP or TRAP	Toorang et al., 2023
TRAP	To evaluate plasma lactate levels after exercise and fatigue and their potential association with antioxidant defense mechanisms	rats	The most sensitive test for determining plasma non-antioxidant capacity following intense exercise is the TRAP assay	Godoy et al., 2022
TRAP	To analyze OS levels and its association with sarcopenia in COPD.	thirty-nine patients with COPD and thirty-five apparently healthy subjects	In COPD, oxidative stress is linked to sarcopenia	Sepúlveda-Loyola et al., 2021
TOSC	To comparatively assess biomarker responses in pelagic and benthic bivalves following Benzo [a]pyrene (B [a]P) exposure, evaluating their utility as bioindicators of PAH toxicity	Benzo [a] Pyrene (B [a]P)	After 7 days of exposure to two doses of benzo [a]pyrene (B [a]P), there was a notable increase in TOSC	Dellali et al., 2021
TOSC	To examine the association between NASH and dietary patterns—specifically Mediterranean diet adherence, food group consumption, and nutrient intake—in individuals with T2D	2026 people with T2D	The NO-NASH model, which is characterized by a greater polyphenol, vitamin, and fiber content similar to the Mediterranean diet model, has a significantly higher antioxidant capacity than the NASH model	Vitale et al., 2022
β-Carotenebleachingassays	To validate the traditional medicinal uses of Lantana rhodesiensis through phytochemical profiling and bioactivity assessment, with specific focus on correlating polyphenolic content to antioxidant and anti-malarial efficacy	Lantana rhodesiensis Moldenke	A positive linear association between the antioxidant activity of all extracts and their phenolic content was found, and leaf extracts demonstrated more antioxidant activity than stem and root extracts	Nea et al., 2021
β-Carotenebleachingassays	To elucidate the structure-activity relationship between hydrophobicity (log P) and biological efficacy of hesperidin derivatives	hesperidin-related compounds	Although to differing degrees, all hesperidin-related substances exhibited concentration-dependent antioxidant activity	Lee et al., 2024
β-Carotenebleachingassays	To characterize the nutritional composition and antioxidant potential of coconut cotyledon through comparative analysis of hot/cold percolated extracts, identifying optimal extraction methods for bioactive compounds	cotyledon of coconut	Significant antioxidant activity was observed in the cotyledons of coconuts	Nyayiru Kannaian et al. (2020)
β-Carotenebleachingassays	To characterize tissue-specific distribution patterns of bioactive metabolites in citrus fruits	citrus fruits	β-carotene bleaching assays can be used to measure antioxidant activity and link it with citrinin concentration	García-Nicolás et al., 2023
β-Carotenebleachingassays	To develop lipase-catalyzed alkyl gallate grafting onto pectin for enhanced biofunctionalization, elucidating how alkyl chain length modulates grafting efficiency, molecular structure, and dual antioxidant/antimicrobial efficacy	the acylated pectins	In the DPPH test and the β-Carotene bleaching assays, acylated pectin shown increased antioxidant activity and was discovered to possess strong antibacterial qualities against *Staphylococcus aureus*and *Escherichia coli*	Zhuang Y et al. (2022)
Chemiluminescentassay	To develop a novel near-infrared chemiluminescent platform for sensitive quantification of urinary total antioxidant capacity in diabetic models, addressing the critical need for simple redox state monitoring in diabetes management	diabetic mice	Semiconducting polymer nanoparticles-manganese (SPN-MnVII)-based chemiluminescence platform design for diabetic mice to detect urine’s overall antioxidant capacity	Wu et al., 2023
Photochemiluminescenceassay	To determine the lipid-soluble ACL and total phenolic content of 14 plant oil industrial by-products, and to evaluate the applicability of different measurement methods to such samples	14 different by-products (flour, couscous and hulled)	PCL’s measurement of fat-soluble substances’ antioxidant capacity and other techniques for assessing antioxidant activity have a strong positive connection	Multescu et al., 2022
Photochemiluminescenceassay	To characterize the chemical profile, determine the optimal extraction method, and evaluate the antioxidant potential of Vinca minor from Dobrogea	Vinca minor macerates	The presence of phenolic chemicals in the maceration solution is strongly correlated with antioxidant activity	Neculai et al. (2023)
Photochemiluminescenceassay	To comprehensively characterize the functional performance, composition, and consumer acceptability of dry probiotic products	Microencapsulated probiotics in dry substrates in snack bars	There was a strong correlation between total phenolic content and antioxidant activity.As TPC dropped over the course of 2 months of storage, antioxidant activity also dropped	Rajagukguk et al. (2022)
Folin-Ciocalteu	To investigate the acute (short-term) metabolic effects of two different doses of a polyphenol-rich curry consumed with white rice	20 healthy men	Twenty healthy men’s glucose homeostasis is improved when they consume curry that is high in (poly)phenol	Haldar S et al. (2019)
Folin-Ciocalteu	To determine whether urinary antioxidant activity correlates with improvements in key cardiovascular risk factors—blood pressure and serum lipid/glucose profiles—in high-risk elderly individuals	573 volunteers	Higher urine (poly)phenol excretion is associated with better cardiovascular health metrics, including blood pressure, body weight, and glucose and lipid levels	Guo et al. (2016)
Folin-Ciocalteu	To evaluate the relationship between urinary total polyphenol excretion and CVRFs in adolescents	1,194 Spanish adolescents	A improved cardiovascular profile was linked to urinary polyphenols. Researchers discover a stronger correlation between phenolic excretion and cardiovascular health in boys than in girls	Laveriano-Santos et al., 2020
Folin-Ciocalteu	To assess the value of polyphenols as a urinary biomarker of an anti-inflammatory diet and their influence on MetS status	543 participants with high CVD risk	Total polyphenol consumption and inflammation are inversely correlated, and measuring the amount of (poly)phenols expelled in urine can be a valid biomarker for an anti-inflammatory diet, particularly in women	Arancibia-Riveros et al., 2023
Folin-Ciocalteu	To evaluate the antihyperlipidemic and antioxidant activities of the total extract and fractions from Anogeissus leiocarpus roots, and identify their potential for treating diabetes-related complications	ICR mice	Anogeissus leiocarpus whole extracts and fractions are rich in polyphenols and have potent antioxidant and anti-hyperlipidemic effects, making them useful for treating macrovascular problems associated with diabetes	Motto et al., 2021
CUPRAC	To compare the chemical composition of the water and hydromethanolic extracts of R. rosea commercial samples in relation to their biological activity	common and lemon verbena leaves	Samples of lemon verbena exhibited greater antioxidant activity than samples of regular verbena, and this activity was positively connected with the amount of phenolic components present	Polumackanycz et al. (2022b)
CUPRAC	To comparatively evaluate the antioxidant and antiradical properties of selected phyto lignans and mammalian lignans using standardized biochemical assays	some phyto lignans mammalian lignans	While enterodiols and enterolactones are less active than phytolignans and conventional antioxidants, phytolignans have a comparatively high level of antioxidant activity	Polat Kose and Gulcin (2021)
CUPRAC	To investigate the compounds in the hexane extract of *Cirsium vulgare*(Savi.) Ten. and to determine the antibacterial, antifungal, and antioxidant activities of different extracts	compounds in the hexane extract of *Cirsium vulgare*	The largest amount of flavonoids and antioxidant activity were found in methanolic extract	Aydın Kurç et al. (2023)
CUPRAC	To comprehensively compare the nutritional profiles, phenolic composition, and antioxidant activities of common verbena and lemon verbena leaves, and evaluate the impact of extraction methods on their bioactive properties	Aqueous and hydro-methanolic extracts of commercial samples of R. rosea	The hydro-methanolic extract of R. rosea contained a higher concentration of phenolic compounds and demonstrated more potent antioxidant and neurobiological effects than the aqueous extract	Polumackanycz et al. (2022a)
CUPRAC	To comparatively evaluate the phenolic content, catechin composition, caffeine levels, and antioxidant properties of 15 commercially available green teas, with specific focus on differentiating ceremonial versus culinary matcha grades, and to assess their cost-effectiveness for antioxidant benefits	fifteen commercially-available green teas	Matcha, gunpowder, and bagged green teas exhibited higher total phenolic content and stronger CUPRAC antioxidant capacities compared to other teas	Meyer et al. (2023)
CUPRAC	To valorize grape seed waste through green extraction of bioactive phenolics, identify the most potent variety, and demonstrate the practical stability of the optimal extract in cosmetic formulations for sustainable product development	Seed Extracts of Marselan, Syrah and Cabernet Franc	Marselan exhibited the highest levels of proanthocyanidin and total phenolic content, coupled with the strongest antioxidant activity	Salem et al. (2022)
FRAP	To evaluate the ACE inhibitory and antioxidant activities of molecular weight-fractionated ultrafiltrates derived from Fucus spiralis protein hydrolysate, and identify optimal fractions for preventing cardiovascular diseases	FSPH-UF	Fr3 was found to have substantial ACE inhibitory activity and the greatest FRAP	Paiva et al. (2017)
FRAP	To produce, fractionate, and evaluate mushroom protein hydrolysates (MPHs) from Agaricus bisporus for their antioxidant potential, identifying optimal enzymatic hydrolysis methods and peptide fractions for functional food applications	MPI	All protein hydrolysates and peptide fractions exhibited concentration-dependent increases. The trypticase hydrolysate demonstrated the highest FRAP value	Kimatu et al. (2017)
FRAP	To evaluate the impact of standard-dose ionizing radiation (25 kGy) on the physicochemical stability and antioxidant properties of solid-state quercetin and rutin, validating their suitability for radiation sterilization	Quercetin and rutin in solid form	Exposure to ionizing radiation does not change the chemical structure of tested flavonoids or their antioxidant properties	Rosiak et al. (2023)
DPPH	To evaluate the antioxidant potential of Ficus religiosa using the DPPH radical scavenging assay and explore its therapeutic applications, particularly in developing metal oxide nanoparticles for combating free radical-related diseases	Seed Extracts of Marselan, Syrah and Cabernet Franc	The antioxidant properties of F. religiosa may be advantageous for treating diseases associated with free radicals	Baliyan et al. (2022)
DPPH	To evaluate the antioxidant activity of various parts of unripe and ripe papaya fruit from the DPPH· kinetics point of view	Papaya fruits	The antioxidant activity of ripe papaya pulp extracts, as determined by the DPPH assay, was found to be 1.2 to 1.4 times greater than that of other ripe papaya extracts	Iordănescu et al. (2021)
DPPH	To evaluate how postharvest processing affects antioxidant activity and secondary metabolite profiles in hops and cannabis inflorescences, and to assess the suitability of DPPH assays for correlating antioxidant activity with these metabolites	MAHD	The antioxidant activity of cannabis was significantly reduced by 60.5% following freeze-drying and MAHD, compared to the pre-freezing samples	Addo et al. (2023)
DPPH	To experimentally determine whether the neuroprotective compound mdivi-1 exhibits direct free radical scavenging activity	Mdivi-1	Mdivi-1 demonstrated antioxidant properties	Bordt et al. (2022)
DPPH	To evaluate the potential of Levilactobacillus brevis strains as starter cultures for lychee juice fermentation, with the aim of enhancing their functionality and nutritional properties	Levilactobacillus brevis strains	The assessment of the antioxidant capacity of Levilactobacillus brevis strains resulted in a significant increase in DPPH scavenging activity	Jin et al. (2023)
DPPH	To investigate the effect of baobab fruit on postprandial glycaemia in healthy adults and to measure its bioactive compounds and antioxidant activity	31 healthy subjects	Baobab fruit extracts notably decreased the incremental glycemic AUC, while also demonstrating significant inhibition of reactive oxygen species and robust antioxidant activity	Rita et al. (2022)
TEAC	To evaluate and compare the antioxidant capacity of traditional homemade fruit vinegars versus commercial vinegars using electrochemicaland TEAC assays, and to correlate these findings with physicochemical properties, production methods, and fruit types	traditional homemade fruit vinegar	The antioxidant activity of all homemade fruit vinegars was at least ten times higher than that of commercial fruit vinegars	Chochevska et al. (2021)
TEAC	To develop and validate a robust machine-learning model for accurate prediction of TEAC, providing a computational alternative to laboratory-based assays	PSO-ELM	PSO-ELM exhibits a remarkable capability in predicting Trolox equivalent antioxidant capacity values and serves as an effective alternative to laboratory data	Elveny et al. (2021)
TEAC	To evaluate cysteine as an anti-greening agent for inhibiting undesirable green trihydroxy benzacridine formation in CGA-lysine reactions, while assessing its impact on antioxidant capacity under alkaline conditions	CGA quinones	The impact of pH on antioxidant capacity was more significant than the addition of cysteine. The greening of chlorogenic acid quinone amine under alkaline conditions may represent a potential anti-greening strategy	Liang and Were (2020)
TEAC	To investigate the synergistic therapeutic potential of combined angiotensin II receptor blocker and IGF-1 replacement in attenuating NASH-associated skeletal muscle atrophy	MCD-fed mice	Treatments involving ARB and IGF-1 enhanced Trolox-equivalent antioxidant capacity and increased levels of antioxidant enzymes	Tanaka et al. (2024)

#### 3.3.10 DPPH

Baliyan S. et al. evaluated the antioxidant capacity of F. religiosa using DPPH and concluded that it possesses antioxidant properties that could be beneficial in treating diseases linked to free radicals (Baliyan et al., 2022). Iordănescu O. et al. discovered that ripe papaya pulp extracts exhibited the highest antioxidant activity measured by DPPH, being 1.2–1.4 times greater than other ripe papaya extracts (Iordănescu et al., 2021). Addo P.W. et al. investigated the relationship between total antioxidant capacity, cannabinoids, and terpenoids in hops and cannabis, finding that the DPPH assay indicated a significant reduction of 60.5% in antioxidant activity of cannabis after freeze-drying and MAHD compared to pre-freezing samples, although no significant change was observed with the FRAP method (Addo et al., 2023). Bordt E.A. et al. reported that Mdivi-1 displayed antioxidant activity in both ABTS and DPPH assays (Bordt et al., 2022). Jin Y. found that testing the antioxidant capacity of Levilactobacillus brevis strains resulted in a significant increase in DPPH and FRAP, alongside a decrease in ABTS antioxidant capacity (Jin et al., 2023). Keyla Rita et al. conducted a randomized controlled clinical trial with 31 subjects, testing antioxidant capacity before and after using FRAP, DPPH, and ABTS, and found that baobab fruit extracts significantly reduced the incremental glycemic AUC while demonstrating considerable suppression of reactive oxygen species and antioxidant activity (Rita et al., 2022).

#### 3.3.11 TEAC

ChochevskaM and team assessed the antioxidant properties of traditionally prepared homemade fruit vinegar through the ABTS^+^• method. Their independent assessments of antioxidant capacity, utilizing the TEAC assay—which is intended to align with electrochemical experiment data—revealed that all homemade fruit vinegars exhibited at least ten times greater antioxidant activity compared to commercial fruit vinegars (Chochevska et al., 2021). ElvenyM et al. discovered that the PSO-ELM model, which is straightforward and precise, effectively predicts Trolox equivalent antioxidant capacity values and can serve as a reliable alternative to laboratory data (Elveny et al., 2021). LiangY et al. assessed antioxidant capacity through the Folin-Ciocalteau and Trolox equivalent antioxidant capacity assays, noting that pH had a more significant impact on antioxidant capacity than the addition of cysteine. Their findings suggest that alkaline chlorogenic acid (CGA) quinone amine greening could be a viable anti-greening strategy (Liang and Were, 2020). TanakaM et al. reported that treatments with ARB and IGF-1 enhanced Trolox-equivalent antioxidant capacity and increased levels of antioxidant enzymes (Tanaka et al., 2024).

## 4 Oxidative stress in various diseases

### 4.1 Alzheimer’s disease

Alzheimer’s disease (AD) involves a range of complex mechanisms and theories, with oxidative stress and its detrimental effects on neurons being fundamental components in all these processes. Under normal conditions,ROS are essential for signaling pathways and transcription activation, and they are closely linked to the different pathogenic processes of AD. However, when ROS levels rise beyond the capacity of antioxidants, they can damage cellular macromolecules, disrupt normal cell functions, trigger mitochondria to produce pro-apoptotic proteins, and ultimately lead to the apoptosis of central nervous system neurons (Schieber and Chandel, 2014). Oxidative Stress and the Amyloid Cascade Hypothesis: ROS significantly contribute to Aβ (amyloid-β)-induced neuronal apoptosis. Impaired mitochondrial function results in the release of more ROS, which activates pro-apoptotic protein production, culminating in nerve cell apoptosis (Bai et al., 2022). Additionally, extracellular amyloid plaques exacerbate the reaction to oxidative stress. Further advancing the pathological progression of AD. Oxidative Stress and the Tau Hypothesis: ROS attacks tau proteins, leading to interactions with CDK and GSK3β kinases, which causes excessive phosphorylation (Lau et al., 2002; Avila et al., 2010). This process hinders the transport of proteasome peroxidase, resulting in increased oxidative stress. Furthermore, ROS stimulation promotes the formation of disulfide bonds in tau proteins, worsening oxidative stress and encouraging tau accumulation (Saito et al., 2021). Oxidative Stress and the Inflammation Hypothesis: Aging and ROS activation lead to the stimulation of microglia. Once activated, these microglia release ROS, inflammatory cytokines, and chemokines (Thakur et al., 2023), initiating a chronic inflammatory response and enhancing the synthesis of cathepsin B protein (Chaney et al., 2019). These events ultimately result in neuronal dysfunction and apoptosis. Oxidative Stress and the Metal Ion Hypothesis: Different types of ROS interact with various metal valences (Iqbal et al., 2014), intensifying neuronal cell apoptosis and contributing to AD pathogenesis.

### 4.2 Diabetes

Oxidative stress not only directly harms islet beta cells but also acts as a signaling molecule that activates various stress-sensitive pathways. This regulation influences the expression of related factors, resulting in beta cell apoptosis or necrosis, decreased insulin secretion, and increased insulin resistance, ultimately contributing to the onset or progression of diabetes (Drews et al., 2010). Impact on Islet β Cell Function: Oxidative stress disrupts the structure of mitochondria in islet β cells, leading to apoptosis and The nuclear transcription factor κB (NF-κB) is activated signaling pathway, which triggers inflammatory responses within the cells (Wronka et al., 2022). Furthermore, oxidative stress can impede the nucleoplasmic translocation of pancreaticoduodenal homeobox factor 1 (PDX-1), impair energy metabolism, and reduce insulin production and release (Duni et al., 2019). Induction of Insulin Resistance: Oxidative stress disrupts the activation of phosphatidylinositol 3-kinase (PI3K) by impairing the phosphorylation of insulin receptors (InsR) and insulin receptor substrates (IRS) (Henriksen et al., 2011). Furthermore, it inhibits the translocation of glucose transporter 4 (GLUT4) (Wu et al., 2016) and damages the cytoskeleton, along with other physiological processes related to insulin signaling. This cascade of events ultimately leads to the development of insulin resistance (Moroz et al., 2008).

### 4.3 Lung cancer

In nearly all cancer types, excessive accumulation of ROS contributes to cancer development by enhancing pro-tumor signaling. There is strong evidence linking elevated ROS levels to cancer progression (Glorieux et al., 2024). Abnormal Cell Signaling Pathways: The epithelial growth factor receptor (EGFR), part of the human epidermal growth factor receptor (HER) family (Yang and Chang, 2018) and transmembrane receptor tyrosine kinase family (Singal et al., 2019), can become abnormal through gene amplification, mutation, or overexpression. This disruption in signaling pathways is a critical factor in tumor formation (Costa et al., 2017; Yewale et al., 2013), particularly in lung cancer, where EGFR plays a vital role (Peifer et al., 2012). DNA Damage: Oxidative stress can inflict DNA damage, hinder DNA repair mechanisms, and promote cell proliferation, facilitating lung cancer development (Stefanou et al., 2022). ROS can directly attack DNA, causing oxidative damage (Taniguchi et al., 2012), including base damage and single-strand breaks, which can cause DNA to mutate and change structurally (Roy, 2014). Additionally, oxidative stress impairs the cell’s ability to repair damaged DNA by affecting related signaling pathways and enzymes, promoting tumor cell proliferation and causing cell cycle arrest, which impacts overall cell proliferation.

### 4.4 Coronary heart disease

Excessive ROS accumulation has been shown to upset the equilibrium between the oxidation and antioxidant systems, leading to oxidative stress, which is a major factor in the development of coronary heart disease. Promotion of Atherosclerosis: Atherosclerosis is a key factor in coronary heart disease, beginning with endothelial cell injury (Xu et al., 2021). Under oxidative stress, nitric oxide’s biological activity diminishes, affecting blood vessel tension and leading to endothelial dysfunction. Damaged endothelium is more prone to lipid accumulation (Zhang et al., 2018), thrombosis, immune cell invasion (Libby, 2021), inflammatory responses (Ribeiro et al., 2010), and abnormal blood flow, all contributing to atherosclerosis. Excessive ROS disrupt the oxidation-antioxidant balance, activate inflammatory cell signals, and induce endothelial cell dysfunction through mitochondria-mediated apoptosis, promoting vascular smooth muscle cell proliferation, interfering with nitric oxide synthesis, and delaying endothelial repair, thereby accelerating atherosclerosis development (Tullio et al., 2013; Zhang et al., 2015).

**FIGURE 1:**
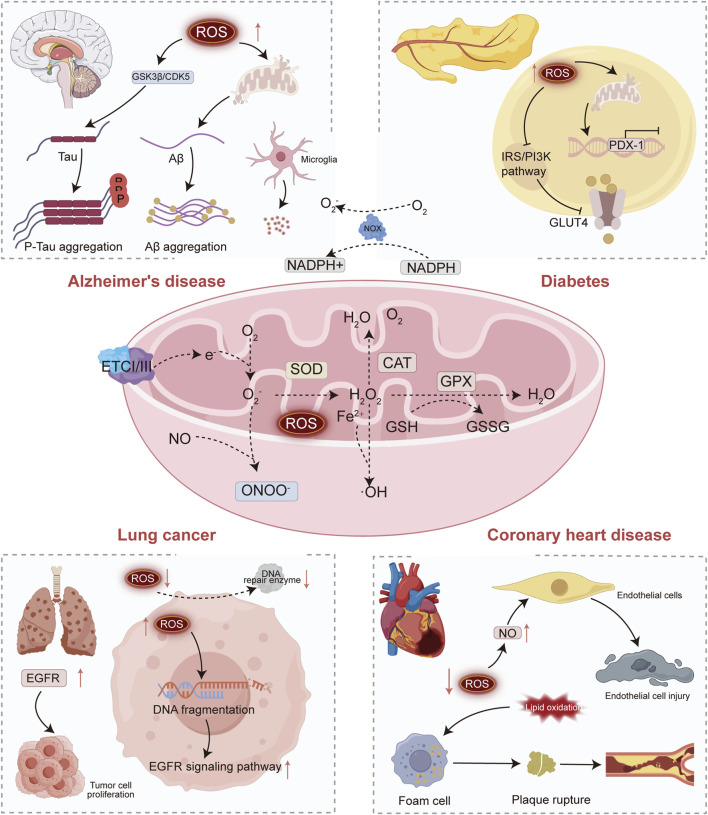
Oxidative stress mechanism and related diseases.

Oxidative stress originates from mitochondrial electron leakage at ETC complexes I/III, producing O_2_- that drive ROS cascades (e.g., H_2_O_2_, ·OH). Cellular protection involves enzymatic (SOD, CAT, GSH-Px) and non-enzymatic (GSH, ALA) antioxidants. A sustained ROS imbalance propagates disease-specific pathology. In Alzheimer’s disease, ROS exacerbate Aβ plaque deposition and tau hyperphosphorylation, inducing neuroinflammation. Diabetes pathogenesis features ROS-mediated β-cell apoptosis and impaired GLUT4 translocation, worsening insulin resistance. Lung cancer progression is fueled by ROS-induced EGFR mutations and DNA damage, while coronary heart disease arises from endothelial dysfunction and LDL oxidation. This framework thereby bridges molecular mechanisms to clinical disease, demonstrating oxidative stress as a unifying axis in multifactorial pathologies. Figure 1 shows the relationship between oxidative stress mechanisms and related diseases.

## 5 Exogenous source of antioxidants with therapeutic potential

At present, there are many types of antioxidants in use, including essential oils (EO) (de Sousa et al., 2023) extracted from various plants, ALA which is used clinically to treat diabetic peripheral neuropathy (DPN), phenolic substances, vitamin C and E, MT, etc. Besides their general antioxidant effects, these antioxidants also play significant roles in specific diseases such as neurological disorders, various tumors, cardiovascular diseases, and endocrine disorders.

### 5.1 Antioxidants for neurological disorders

Neurological diseases such as Alzheimer’s disease and epilepsy, due to their complexity and severity, present numerous challenges in clinical treatment. Due to the crucial role of oxidative stress in it, the use of antioxidants becomes possible. ALA is a commonly used drug in clinical practice for treating DPN. Its ability to protect nerve function and alleviate clinical symptoms has been widely recognized (Fasipe et al., 2023; Oraebosi et al., 2022). In addition,it has also been implicated in other neurological disorders (Khan et al., 2022). A randomized controlled trial indicated that ALA improves psychopathology and lessens oxidative stress in schizophrenic patients who are treatment-resistant schizophrenia (TRS) (Mishra et al., 2022). In epilepsy patients, repeated seizures and prolonged medication can lead to reactive oxygen species production, causing neuronal damage. ALA, as an antioxidant, binds strongly to MnSOD protein, enhancing drug efficacy and inhibiting seizures (Javaid et al., 2022). In animal experiments, ALA also demonstrated a special therapeutic effect on neurological disorders. Animal studies have shown that ALA can mitigate aggressive behavior in mice, suggesting its relevance for psychological disorders (Karim et al., 2023). Studies also have demonstrated that ALA can reduce lipid peroxidation, protein carbonylation, and advanced oxidized protein products induced by the antiepileptic drug valproic acid (VPA) (Turkyilmaz et al., 2020). Additionally, ALA administration in rats post-spinal cord injury has been shown to decrease antioxidant formation (Ercan et al., 2021). Vitamins C and E are natural antioxidants found in the human body. In clinical practice, they are often used as adjunctive medications and work together with other drugs. Research indicates that antioxidants like vitamin E can prevent lipid peroxidation and iron accumulation in neurodegenerative conditions associated with brain iron accumulation (NBIA), partially improving the physiological pathology of affected cells (Suárez-Carrillo et al., 2023). Vitamin C has been shown to alleviate cisplatin-induced neuropathy by increasing levels of heat shock protein-70 and nerve growth factor while reducing inflammation and oxidative stress (Pala et al., 2022).

Apart from the common ALA and vitamins in practice, other antioxidants, although not yet widely used, also show potential in treating neurological diseases. The essential oil from Alpinia zerumbet (EOAZ) has shown effects similar to olanzapine in mitigating schizophrenia-like symptoms induced by repeated ketamine administration, with fewer side effects (de Araújo et al., 2021). Cachrys sicula L. (Apiaceae) EO effectively inhibits key enzymes associated with Alzheimer’s disease, particularly butyrylcholinesterase (BChE) (Tahar et al., 2022). Additionally, phenols have therapeutic potential for AD, stroke, depression, and Parkinson’s disease (Liu et al., 2023). MT and its metabolites regulate various sirtuin pathways related to apoptosis, proliferation, metastasis, autophagy, and inflammation in stroke (Azedi et al., 2022). Its neuroprotective effects have been supported by numerous studies (Sobhani et al., 2023). However, with the exception of a few cases, most of these antioxidants have not been put into clinical use. The complexity of the components (such as EO), the diversity of their effects (such as hormones), and other factors all pose limitations to its treatment of neurological diseases. More clinical trials, rather than just animal experiments, are needed to further confirm its exact efficacy.

### 5.2 Antioxidants for tumors

Malignant tumors are severe diseases caused by the uncontrolled and abnormal proliferation of cells. How to accurately kill cancer cells and prevent the tumor from continuing to grow has always been a key issue in cancer treatment. And antioxidants have shown certain potential in this regard. Ganoderma EO demonstrated anti-tumor properties, inducing apoptosis in K562 cells and inhibiting cell proliferation by blocking the cell cycle in the S phase (Wang et al., 2023). Prangos pabularia EO (20 μg/mL) exhibited significant cytotoxicity against human lung adenocarcinoma cells (A549) (56.12%) (Banday et al., 2022). The primary component of Inula viscosa EO, (E)-Z-farnesyl acetone, acts as a functional inhibitor of VEGF activity, potentially hindering abnormal angiogenesis in conditions like tumors and diabetic retinopathy (Nadia et al., 2022). Furthermore, turmeric EO exhibits a range of effects, including anti-cancer, anti-inflammatory etc. (Orellana-Paucar, 2024). Polyphenols can exert antioxidant and immunomodulatory effects by targeting NF-κB, thereby inhibiting tumor development (Guan et al., 2022). Phenols derived from extra virgin olive oil (EVOOE) have shown anti-proliferative effects on bladder cancer cells (Spagnuolo et al., 2022). Certain trace elements, such as Se, not only participate in antioxidant processes as oxidoreductase components but also exhibit anti-cancer and immune-regulating effects (Huang et al., 2022; Hariharan and Dharmaraj, 2020; Dogaru et al., 2023). Compared with the highly toxic radiotherapy and chemotherapy, antioxidants are undoubtedly a better treatment option for patients. However, their effectiveness still needs to be explored over a longer period of time.

### 5.3 Antioxidants for cardiovascular diseases

We know that the elevation of many metabolites can cause damage to the cardiovascular system, such as low-density lipoprotein and glucose. Antioxidants’ protective effect on the cardiovascular system is often achieved by regulating metabolism. In animal studies, administering laurel and myrtle EOs to rats resulted in weight loss and improvements in lipid profiles (cholesterol, triglycerides, LDL-C, and VLDL-C) and atherosclerotic markers by regulating liver lipid metabolism (Odeh et al., 2022). Research has shown that various polyphenols can provide cardiovascular protection by binding to lipoproteins and preventing their oxidation at both lysosomal/inflammatory pH (5.2) and physiological pH (7.4) (Tung et al., 2020). An overview of the above antioxidants, along with their targets and clinical status, is shown in Table 3. Their primary mechanism involves reducing circulating cholesterol, binding to LDL particles, and enhancing systemic antioxidant activity (Ahmadi et al., 2022). Zhang et al. developed a compound by combining the anti-inflammatory and antioxidant polyphenol EGCG with Fe (3+) and atorvastatin, resulting in EGCG-FE-ATV, which demonstrated significant anti-inflammatory, antioxidant, and lipid-lowering effects, along with good biocompatibility and biosafety (Zhang et al., 2023). However, not all antioxidants have a definite effect on the cardiovascular system. The impact of certain phenols on blood lipids may be minimal, as seen with tocotrienol (Zuo et al., 2020). In terms of lipid metabolism, α-tocopherol and ascorbic acid are not effective in inhibiting ferritin oxidation of LDL at lysosomal pH (Ojo and Leake, 2021). This might explain why large-scale clinical trials using these vitamins failed to demonstrate any protective effect against cardiovascular diseases. Regarding glucose metabolism, extracts from Ononis alba Poir L. (Fabaceae) EO showed superior inhibition of α-amylase compared to acarbose (Zaak et al., 2022). In animal studies, MT has been shown to mitigate oxidative stress, apoptosis, and cardiac dysfunction induced by high glucose and STZ by modulating the AMPK/SIRT1 signaling pathway (Wang et al., 2021).

**TABLE 3:** Antioxidants and their associated targets, pathways, diseases.

Antioxidants	Action target or pathway	Targeted disease	Clinical status
Essential oil	NO interaction pathway Glycometabolic pathway Lipid metabolic pathway Apoptosis pathway VEGF pathway BChE	Asthma Tumor Diabetes mellitus Atherosclerosis Alzheimer’s disease	Clinical use (mainly as an adjunctive treatment)
Alpha lipoic acid	The lipid peroxidation pathway Protein carbonylation pathway Neuron MnSOD	Complications of diabetes Treatment-resistant schizophrenia Psychological disorders Epilepsy Kidney disease Infertility	Clinical use (such as DPN)
Phenols	The NF-κB pathway Cytokines and nitric oxide Lipoprotein	Alzheimer’s disease Stroke Depression Parkinson’s disease polycystic ovary syndrome Hyperlipidemia Tumor	Clinical use
Vitamins C and E	Lipid peroxidation pathway Iron accumulation pathway Nerve growth factor	Neurodegeneration with brain iron accumulation	Clinical use (mainly as an adjunctive treatment)
Trace elements	SOD etc.	Tumor etc.	experimental
Melatonin	Sirtuins pathway AMPK/SIRT1 signaling pathway Free radical	cardiac dysfunction Stroke	experimental

### 5.4 Antioxidants for other diseases

In other diseases, antioxidants also play a significant role. Numerous studies have highlighted the antioxidant properties of various essential oils (Zhang et al., 2022; Bungau et al., 2023). Additionally, essential oils serve multiple functions. For instance, virgin coconut EO can alleviate asthma symptoms by addressing peribronchial inflammation, epithelial hyperplasia, smooth muscle thickening, and excessive contraction through oxidative stress and its interaction with the nitric oxide pathway (Vasconcelos et al., 2020). Oregano EO is recognized as a natural antimicrobial agent with potential to combat antimicrobial resistance (AMR) globally (Walasek-Janusz et al., 2024). A review identified ALA’s potential renal protective mechanisms in various kidney injury models, including diabetic nephropathy and sepsis-induced kidney injury (Kamt et al., 2023). Furthermore, ALA has therapeutic potential in diabetic retinopathy, dry eye disease, and systemic sclerosis (Ajith, 2020; Xie et al., 2022). Notably, studies in rodents have indicated that ALA can restore sperm function and address infertility (Naderi et al., 2023). Phenols are common antioxidants that play an anti-inflammatory role by regulating lymphocyte and macrophage activity through the modulation of cytokine and nitric oxide release (Pisoschi et al., 2024). Furthermore, phenolic compounds also have therapeutic potential for polycystic ovary syndrome (PCOS) (Sarmadi et al., 2023). MT, a hormone secreted by the pineal gland, possesses strong neuroendocrine immunomodulatory properties and free radical scavenging abilities. Certain amino acids and peptides can counteract intra-lipid peroxidation (Hajieva et al., 2023) and may have implications for treating non-alcoholic fatty liver disease, diabetic complications, and COVID-19 (Kuerban et al., 2020; Wu et al., 2023). However, at present, most of these applications are still at the stage of animal experiments and there is a long way to go before they can be applied in practice.

## 6 Conclusion

Oxidative stress, defined as an imbalance between the production of reactive oxygen species ROS and antioxidant defenses, plays a pivotal role in the pathogenesis of major diseases, including cardiovascular disorders, diabetes, cancer, and neurodegenerative conditions. ROS, as natural metabolic byproducts, have been shown to damage nucleic acids, proteins, and lipids through mechanisms such as DNA mutations, lipid peroxidation, and altered post-translational modifications, ultimately compromising cellular integrity. Prolonged oxidative conditions have been shown to exacerbate these effects, disrupting critical pathways such as apoptosis, autophagy, and fibrosis. For instance, mitochondrial ROS activate the NLRP3 inflammasome to drive pyroptosis, while TGF-β and ROS form a self-amplifying loop that accelerates fibrotic progression. Furthermore, mitochondrial oxidative stress has been demonstrated to induce ferroptosis via the NRF2-ARE pathway, thereby emphasizing the multifaceted role of ROS in disease mechanisms.

However, controversies persist regarding the net impact of ROS modulation. While ROS scavenging is beneficial in chronic diseases like diabetes (Zhang et al., 2020), excessive antioxidant supplementation may disrupt redox homeostasis, as evidenced by increased cancer risk in vitamin E trials (Jiang, 2024). Moreover, low-level ROS induction via exercise (“mitohormesis”) enhances endogenous defense systems (Kang et al., 2021), contrasting with pathological ROS cascades. This duality necessitates context-specific therapeutic strategies—for instance, pro-oxidant approaches in cancer versus antioxidant interventions in neurodegeneration (Poprac et al., 2017). Therefore, engaging in a more thorough discussion of the controversial or contradictory data arising in the field of oxidative stress research will help to develop a comprehensive perspective. Despite advances in detection methods, there are still significant limitations in terms of reproducibility and clinical applicability. Key challenges include a lack of specificity, where non-specific reactions (e.g., interference from ascorbic acid in the Folin-Ciocalteu assay) can lead to an overestimation of antioxidant capacity; issues with sensitivity due to kinetic discrepancies (e.g., confounding stoichiometry in ORAC resulting from area-under-the-curve integration) and inherent probe limitations (e.g., the pH dependency of fluorescein); and inadequate standardization, as heterogeneous protocols (e.g., the use of arbitrary timepoints in the TEAC assay) impede reliable cross-study comparisons.

Antioxidants, which include both enzymatic (e.g., SOD, CAT) and non-enzymatic agents (e.g., ALA, polyphenols), counteract ROS by restoring redox balance and modulating inflammatory and apoptotic pathways. Phenolic compounds exhibit dual functionality, inhibiting lipoprotein oxidation and suppressing NF-κB-mediated inflammation. In contrast, essential oils target VEGF and apoptosis across a range of disease models. Notwithstanding their considerable potential, challenges such as achieving therapeutic concentrations *in vivo* and tissue-specific delivery impede clinical translation. Nanotechnology-driven strategies effectively tackle fundamental challenges in antioxidant therapy. Liposomal encapsulation significantly enhances pharmacokinetic profiles and enables site-specific accumulation of antioxidants, exemplified by quercetin delivery for atherosclerosis management (Luo et al., 2022). Furthermore, polymeric nanoparticles facilitate blood-brain barrier penetration and central nervous system (CNS) delivery; notably, SOD-loaded PLGA nanoparticles mitigate oxidative neuronal death (Biswas et al., 2025). Mitochondria-targeted antioxidant conjugates further illustrate the precision achievable through these nanoscale interventions (Cheng et al., 2019). Nanocarriers, including liposomes, polymer nanoparticles, inorganic nanoparticles, and organic/inorganic hybrid nanoparticles, represent a promising strategy for enhancing drug delivery. This approach aims to improve therapeutic efficacy while reducing side effects. The employment of detection methodologies, encompassing ORAC, DPPH, and FRAP assays, facilitates the establishment of standardized measures of antioxidant capacity. Concurrently, the utilization of advanced platforms, such as nanoparticle-based chemiluminescence, enables the real-time analysis of biomarkers. Nevertheless, it is imperative to harmonize these methodologies across studies to ensure reproducibility and clinical relevance.

Translational research must prioritize the integration of molecular insights with therapeutic innovation. Combinatorial strategies that target the generation of ROS, such as NADPH oxidase inhibitors, and the depletion of antioxidants, including glutathione precursors, may yield synergistic benefits. To this end, the necessity for longitudinal studies has been underscored, particularly in aging populations where there is a concomitant decline in NRF2-mediated defenses. The application of personalized approaches informed by tissue-specific redox dynamics—such as mitochondrial stress in cancer or ROS-TGF-β interactions in fibrosis—has the potential to refine interventions. For instance, the disruption of ferroptosis pathways or ROS-TGF-β feedback loops has the potential to impede the progression of the disease.

Despite the advances made, limitations remain. Secondary oxidative stress is a common occurrence in disease, often reducing the efficacy of standalone antioxidant therapies. The process of aging has been shown to diminish the efficacy of NRF2 activation, thereby complicating treatment outcomes for elderly patients. A review of global health data underscores the gravity of the situation: cardiovascular diseases have become the leading cause of global mortality, accounting for nearly 18 million deaths annually (Roth et al., 2020). The total economic burden of cardiovascular diseases is estimated at US$177.5 billion per year (GBD 2019 Diseases and Injuries Collaborators, 2020). According to the World Health Organization’s International Agency for Research on Cancer, 19.29 million new cancer cases and 9.7 million cancer-related deaths were recorded globally in 2022 (Bray et al., 2024). Studies project that cancer will incur US$25 trillion in economic losses worldwide between 2020 and 2050 (Chen et al., 2023). The global prevalence of diabetes continues to rise, with 536.6 million affected individuals in 2021; this figure is projected to reach 783.2 million by 2045. Healthcare expenditures related to diabetes reached US$966 billion in the same year and are expected to rise to US$1.054 trillion by 2045 (Sun et al., 2022). Among populations aged 65 years and older, Alzheimer’s disease exhibits a global prevalence of 6.20%, with incidence rates doubling every 5 years. Direct medical costs associated with Alzheimer’s disease totaled US$1.3 trillion in 2022 and are predicted to exceed US$2.08 trillion by 2050 (Wong, 2020). Therefore, it is urgent to adopt oxidative stress-targeted therapies to alleviate the social and economic burden.

In summary, oxidative stress is a core disease mechanism and promotes interdisciplinary efforts to coordinate diagnostic, therapeutic, and mechanistic research. The future agenda entails the standardization of testing protocols, the development of combination therapies, and the advancement of personalized approaches grounded in tissue-specific redox biology. Addressing these challenges will translate redox science into clinical solutions and ultimately reduce the global burden of oxidative stress-related diseases. Future research endeavors should encompass a comprehensive investigation spanning from preclinical studies to clinical trials. Such efforts may advance along three critical dimensions: elucidating the precise underlying mechanisms, characterizing the dynamic behavior of biomarkers, and addressing patient heterogeneity. Priority should be given to selecting therapeutic targets supported by robust preclinical evidence, establishing a systematic biomarker framework, and stratifying potential beneficiaries based on oxidative stress profiles to facilitate individualized and precise treatment strategies. Evaluations should be conducted across various clinical trial phases-including Phase I, Phase II, Phase IIb, and Phase III-to tailor antioxidant therapies to specific disease subtypes, ranging from simple antioxidant interventions to combination therapies. Methodologically, employing randomized controlled trials with high evidentiary standards, longitudinal cohort studies, and umbrella platform trial designs is recommended. Furthermore, the integration of advanced technologies, such as artificial intelligence, holds promise for accelerating the advancement of precision medicine within this domain.

## Glossary

AAPH: 2,2'-Azobis(2-amidinopropane) dihydrochloride
AD: Alzheimer’s Disease
ALA: Alpha-Lipoic Acid
AMPK/SIRT1: AMP-activated protein kinase/Sirtuin 1
AUC: Area Under the Curve
BChE: Butyrylcholinesterase
CAT: Catalase
CGA: Chlorogenic acid
CoQ10: Coenzyme Q10
COPD: Chronic Obstructive Pulmonary Disease
CVD: Cardiovascular Disease
CUPRAC: cupric reducing antioxidant capacity
DPPH: 2,2-diphenyl-1-picrylhydrazyl
EGCG: Epigallocatechin gallate (corrected from ECG)
EGFR: Epidermal Growth Factor Receptor
EOs: Essential Oils
ETC: Electron Transport Chain
FRAP: ferric reducing antioxidant power
GLUT4: Glucose transporter 4
GSH-Px: Glutathione peroxidase
**H**_**2**_ **O**_**2**_: Hydrogen peroxide
HDL: High-Density Lipoprotein
HNC: Head and Neck Cancer
HS-GC: Headspace Gas Chromatography
KMBA: α-Keto-γ-methylthiobutyric acid
LDL: Low-Density Lipoprotein
MnSOD: Manganese-dependent Superoxide Dismutase
MS: Multiple Sclerosis
MT: Melatonin
NF-κB: Nuclear factor kappa B
NO: Nitric oxide
NOX: NADPH oxidase
**O**_**2**_^**−**^: Superoxide anion
**ONOO** ^**−**^: Peroxynitrite
ORAC: oxygen radical absorbance capacity
OS: oxidative stress
PCOS: Polycystic Ovary Syndrome
PCL: Photochemiluminescence
PDX-1: Pancreaticoduodenal homeobox factor 1
Prx: Peroxiredoxin
ROS: Reactive Oxygen Species
RTT: Rett Syndrome
SOD: Superoxide dismutase
TEAC: Trolox-Equivalent Antioxidant Capacity
TOSC: total oxidant scavenging capacity
TRAP: total radical-trapping antioxidant Parameter
Trx: Thioredoxin
T2D: Type 2 Diabetes Mellitus
VEGF: Vascular Endothelial Growth Factor
VPA: Valproic acid
OH: hydroxyl radical
